# Does persistent snowpack inhibit degradation of fecal stress indicators?

**DOI:** 10.1093/conphys/coy071

**Published:** 2018-12-20

**Authors:** Grace L Parikh, Christopher R Webster, John A Vucetich, John J Durocher, Joseph K Bump

**Affiliations:** 1 School of Forest Resources and Environmental Science, Michigan Technological University, Houghton, MI, USA; 2 Department of Biological Sciences, College of Sciences and Arts, Michigan Technological University, Houghton, MI, USA; 3 Department of Fisheries, Wildlife and Conservation Biology, College of Food, Agricultural and Natural Resources Sciences, University of Minnesota, St. Paul, MN, USA

**Keywords:** corticosteroids, fecal glucocorticoids, non-invasive, sample degradation, stress

## Abstract

Physiological stress in wildlife can be a useful indicator of a population’s response to environmental factors. By using non-invasive endocrinological techniques, such as fecal sampling, potential confounding factors associated with the stress of capture can be avoided. A potential drawback of fecal sampling, however, is degradation of samples which may produce aberrant measurements of fecal glucocorticoid metabolites. In vertebrates, glucocorticoids, such as corticosterone, become elevated in response to stress. We sought to gauge the reliability of measurement of fecal glucocorticoid metabolites from white-tailed deer (*Odocoileus virginianus*) fecal samples exposed to a temperate winter with substantial snow cover and cold temperatures for up to 90 days, by repeatedly subsampling fecal samples every 10 days and performing a corticosterone enzyme-linked immunosorbent assay (ELISA). Measurements of fecal glucocorticoid metabolites at 10 days were consistent with initial measurements, after which (20 days) they became aberrant following a period of thawing. Consequently, glucocorticoid metabolite levels in feces appear to remain stable under ambient conditions if temperatures remain below freezing at least for 10 days. While it’s possible that samples may remain useful beyond this time frame based on previous laboratory studies of samples stored in a freezer, further work is needed to determine how samples weather *in situ* under extreme cold (e.g. Arctic) or periods of partial thawing.

## Teaser

Stress can be measured non-invasively for free-ranging animals using fecal hormone metabolites. However, fecal samples subject to warm temperatures, wet conditions, or freeze-thaw cycles are liable to degrade. Our results suggest that samples deposited on a persistent snowpack may provide reliable measurements for up to 10 days.

## Introduction

Conservation physiology has become an important part of understanding the health of wildlife populations. For example, non-invasive endocrinological techniques allow researchers to monitor response to stressors, such as human activity, predation or weather conditions. In addition to gauging stress responses, these techniques can provide insight into an animal’s reproductive status and condition that may affect population recruitment (Wikelski and Cooke, 2006). By understanding the stress response associated with various environmental factors, more effective management decisions are potentially possible.

The physiological response to stress in vertebrates leads to increased production of catecholamines and stimulation of the hypothalamic-pituitary axis (HPA), which induces production of glucocorticoids from the adrenal cortex, such as cortisol and corticosterone (Sheriff et al., 2011). Glucocorticoids lead to mobilization of energy reserves necessary to mitigate a stressor (e.g. outrun a predator). Although this is adaptive in the short-term, long-term elevation in glucocorticoids can alter HPA function and jeopardize an individual’s fitness through immunosuppression and/or reduced reproductive success (Dantzer et al., 2014; Barelli et al., 2015).

As glucocorticoids are metabolized, the resulting metabolites are excreted in feces. The metabolites represent glucocorticoids metabolized over recent days or weeks providing a composite index of stress (Millspaugh and Washburn, 2004; Dantzer et al., 2014). Fecal measurements provide a non-invasive alternative to blood-based measurements for population level studies, which have the advantage of avoiding the stress of capture and being cost-effective (Millspaugh and Washburn, 2004; Dantzer et al., 2014). Non-invasive techniques may also lower cost per sample and facilitate larger sample sizes.

Although fecal glucocorticoid metabolites are a useful index of an animal’s condition, there are some caveats to using this technique. Specifically, deposition environment may influence glucocorticoid metabolite measurements (Wilkening et al., 2016). Ambient conditions are liable to be less stable; warm temperatures, humidity and rain have been associated with sample degradation and aberrant measurement values (Washburn and Millspaugh, 2002; Wilkening et al., 2016). Laboratory experiments involving simulated freeze-thaw exposure of fecal pellets led to aberrant measurements in comparison to initial measurements (Washburn and Millspaugh, 2002). In contrast, fecal samples stored in a freezer provided stable glucocorticoid metabolite measurements for up to a year (Beehner and Whitten, 2004). However, most evidence regarding degradation of fecal glucocorticoid metabolites comes from short-term studies (1–3 weeks) or field experiments under moderate weathering conditions (Washburn and Millspaugh, 2002; Evans et al., 2013; Wilkening et al., 2016). Given that the utility of fecal glucocorticoid metabolites depends on the ambient environment into which samples are deposited, there is a clear need to characterize degradation dynamics under a range of climatic conditions and weathering situations. We sought to assess effects of ambient conditions on fecal sample weathering in a region with consistent sub-freezing temperatures and substantial snow cover during the winter. Deep persistent snow packs are characteristic of ~50 % of the northern hemisphere where ungulates range (Armstrong and Brodzik, 2001). We hypothesized that samples deposited into a persistent snowpack would remain viable as long as freezing conditions persisted.

## Study system and methods

Samples were collected from a winter deer-yarding complex near Prickett Lake (Houghton County, Michigan, USA, 46.7°N, 88.7°W), located on an outlying tract of the Michigan Technological University Ford Forest in Michigan’s Upper Peninsula. Mean annual snowfall in Houghton County is 6.86 m ± 1.14 m (National Weather Service, 2018). Between 2006 and 2017, mean annual snowpack depth was 0.37 m (from 15 November—15 April), and exceeded this depth an average of 76 days per year (National Operational Hydrologic Remote Sensing Center, 2004), calculated using the Snow Data Assimilation System (SNODAS). The study area is characterized by persistent white-tailed deer (*Odocoileus virginianus*) use, with annual over-wintering deer densities of up to 97 deer/km^2^ (Murray et al., 2014). The overstory at this site was predominantly eastern hemlock (*Tsuga canadensis*), which provides high-quality winter habitat for white-tailed deer (Mladenoff and Stearns, 1993; Morrison et al., 2003). In the northern Great Lakes region, white-tailed deer forage over a wide range in warm months, and congregate in conifer stands during winter, due to forage availability and reduced snow depth (Verme, 1973; Doepker et al., 1994), which facilitates predator avoidance (Nelson and Mech, 1981).

We collected 15 fecal samples from white-tailed deer in January 2017 by following their tracks in the snow. To ensure that samples were recently deposited, we collected samples within a day of the most recent snowfall. To reduce the likelihood of repeatedly sampling the same individual, each sample was collected from a different set of tracks, spaced sufficiently far apart (~100 m or more) as to likely represent tracks from a different individual.

For simulated weathering, we took a subsample for immediate hormone extraction and assay, and placed the remaining samples in an area outside, with minimal human disturbance and exposure to ambient conditions. Each sample (individual pellet group) was divided into nine subsamples, consisting of two fecal pellets. Subsamples were placed in fine-mesh bags to facilitate recovery and minimize disturbance to the exposure area and other samples. Subsample placement within the exposure area was randomized and occurred within 48 h of field collection. Because fecal masses exhibit heterogeneity in glucocorticoid metabolite distribution (i.e. hotspots in a mass) (Millspaugh and Washburn, 2003), we thoroughly mixed the sample prior to subsampling. Every 10 days, we removed one subsample per individual from the snow for assay, for a total of 10 measurements per individual including time zero.

We used the enzyme-linked immunosorbent assay (ELISA) technique to measure glucocorticoid metabolites in a fecal sample extract. To prepare the sample, we first dried subsamples at 60°C per Palme et al. (2013) for 8 h prior to grinding. Then we used a vortex to mix 0.2 g of the homogenized material with 2 ml methanol. Next, we decanted the slurry and placed it in a centrifuge for 20 min at 2200 rpm (Creel et al., 2002). Samples were stored at −80°C until immunoassays were performed.

Glucocorticoid metabolite measurements were done using ELISA corticosterone kits from MP Biomedicals (Appendix 1). The ELISA assays were performed by placing the fecal extract on a microplate treated with corticosterone antibodies. A second antibody was introduced. Both of these antibodies bind to sites on the corticosterone molecule before an enzyme is introduced to create a product allowing for the concentration of corticosterone to be measured spectrophotometrically (Guyton and Hall, 2006). We followed the protocol recommended by MP Biomedicals for the ELISA corticosterone assay. Standard curves were created from six standards (15–2250 ng/ml). The interassay coefficient of variation was 13%, and the intra-assay coefficient of variation was 6%.

It is important to note that in these assays, there is substantial cross-reactivity, and it is likely that antibodies in these immunoassays detect metabolites of both cortisol and corticosterone. Antibodies may not be able to distinguish between the two glucocorticoids (Möstl et al., 2005; Koren et al., 2012). For white-tailed deer, cortisol is the dominant glucocorticoid, and ratios of cortisol:corticosterone in plasma are 5:1 or higher (Bubenik et al., 1975; Koren et al., 2012).

Snowfall data were obtained from the National Operational Hydrologic Remote Sensing Center, for dates 1 Febraury–2 May 2017 (2004). Temperature data were obtained from the National Oceanic and Atmospheric Administration’s National Data Buoy Center (2017), based on the Portage Canal station, for dates Feb 1 to May 2, 2017.

## Statistical analysis

We tested the expectation that glucocorticoid metabolite concentration would vary with sample exposure time using a repeated measures mixed model, with glucocorticoid metabolite concentration as the response variable, and snow depth (m), sample exposure time (days since placement), and 10-day maximum temperature (°C) as fixed effects, and individual deer as a random effect. We fit this model using the (R Core Team, 2017) package nlme (Pinheiro et al., 2017) and assessed model quality based on *R*^2^ using the (R Core Team, 2017) package MuMIn (Barton, 2018). To determine the point at which samples degraded and showed significant differences in glucocorticoid metabolite concentration from the initial measurements, we fit a second model with individual deer as a random effect and sample exposure time as a categorical variable. Differences between exposure times were then compared post hoc using an all pair-wise Tukey’s honestly significant difference (Keppel, 1991).

## Results

**Figure 1:**
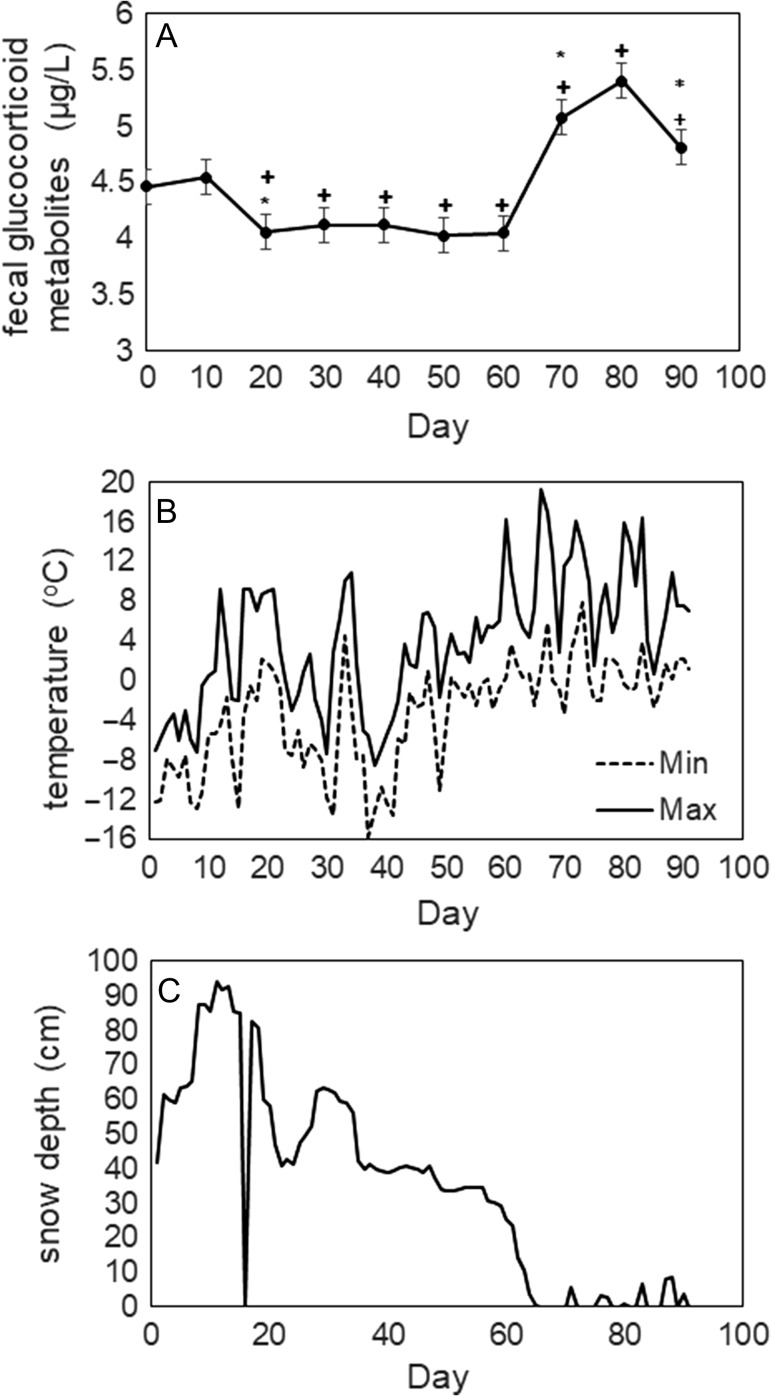
(A) Mean fecal glucocorticoid readings for 15 white-tailed deer fecal samples after 90 days of weathering. A plus sign (+) denotes a statistically significant difference between that subsample and the original measurement, and an asterisk (*) denotes a statistically significant difference between that subsample and the previous one. (B) Daily maximum temperature (solid line) and minimum temperature (dashed line) at study site. (C) Daily snow depth at exposure site for duration of study.

Winter 2017 was marked by periodic freezing and thawing during the study prior to spring thaw (Fig. 1). Just prior to Day 20 of the study, temperatures spiked and snow melted completely at the exposure site, which was underlain by concrete; however, snow remained adjacent to the exposure area. Snow accumulated again around Day 30, and temperatures dropped back below freezing. When temperatures remained below freezing, glucocorticoid metabolite readings appeared stable (Fig. 1). Aberrant measurements were associated with decreases in snow depth associated periods of thawing, especially at the end of the study during spring thaw (Fig. 1).

A repeated measures mixed-model, with glucocorticoid metabolite concentration as the response variable, and snow depth, sample exposure time and 10-day maximum temperature as fixed effects, and individual deer as a random effect (*R*^2^ = 0.37, *P* < 0.001) found that both snow depth (*P* < 0.001) and sample exposure time (*P* = 0.03) were weakly negatively associated with glucocorticoid metabolite concentration. However, 10-day maximum temperature was not associated with glucocorticoid metabolite concentration (*P* = 0.37). To further investigate the influence of exposure time on glucocorticoid metabolite concentration, we fit a second model (*F *= 12.91_(1, 146_), *P* < 0.001) with exposure time as a categorical variable and individual deer as a random effect and contrasted differences at each time period to the initial concentration at exposure time 0 (i.e. placement in the weathering area) and subsequent concentrations. Post hoc comparisons using Tukey’s honestly significant difference showed that there was no difference in glucocorticoid metabolite concentration between 0 and 10 days of exposure (*P* = 0.99). The difference in glucocorticoid metabolite concentration between 0 and 20 days of exposure became significant (*P* = 0.04) (Fig. 1). A difference was also observed between 10 and 20 days of exposure (*P* = 0.004), but not between subsamples at 30–50 days exposure (*P* ≥ 0.990). Difference again appeared between 60 and 70 days of exposure (*P* < 0.001) and 80 and 90 days of exposure (*P* < 0.001). No other significant differences were observed (*P* > 0.05).

## Discussion

Our results indicate that glucocorticoid metabolite measurements from fecal pellets may remain stable when deposited into an environment with persistent sub-freezing temperatures and no snowmelt. This is in contrast to regions with more moderate climate conditions because both gut microbes and environmental microbes degrade fecal masses, beginning at deposition (Möstl et al., 1999; Parnell et al. 2015) and their activity may be accelerated or inhibited by ambient conditions (Mesa-Cruz et al., 2014). In warm, wet climates, fecal samples are likely to degrade in less than a day, as a consequence of high microbial and enzymatic activity. Cooler, wet climates produce similar trends in destabilization of samples because of the influence of moisture on microbial and enzymatic activity (Washburn and Millspaugh, 2002). In hot, dry climates, samples are likely to be stable for up to 5 days, due to inhibited microbial activity (Mesa-Cruz et al., 2014). Consequently, in regions with deep persistent snow packs and sub-freezing temperatures, fecal glucocorticoid metabolites may provide a reliable measure of animal condition because freezing temperatures inhibit microbial and enzymatic activity.

Our results suggest that extended exposure of fecal samples may lead to degradation as a result of fluctuations in ambient temperatures and periods of thaw. For example, fecal glucocorticoid metabolite concentrations began to significantly deviate from the initial concentration between 10 and 20 days, a period that experienced a pronounced warm up to 9.3°C. Sample degradation under ambient conditions was also observed beyond 1–2 weeks by Wilkening et al. (2016) across the western USA, under a range of temperature and moisture conditions. Although glucocorticoid metabolite concentration deviated significantly from the original values beginning at 20 days, at Days 30–60, concentration did not change significantly with these consecutive subsamples. This period was marked by consistent snow cover and minimum temperatures that remained below freezing suggesting that, in our case, degradation was associated with thawing. Our finding of stable glucocorticoid metabolite measurements in samples exposed to consistent winter conditions is similar to results obtained from Beehner and Whitten (2004), where fecal samples were stored in a freezer for over a year and produced stable measurements. However, because ambient conditions under normal field conditions and in our study were not as stable, they did not provide the same degree of sample preservation as a freezer.

Freeze-thaw periods produced aberrant readings, but the direction of these changes in concentration were not consistent, except during spring thaw, when glucocorticoid metabolite concentration increased substantially. The increase during spring thaw could be attributable to increased moisture and warmer temperatures. Together, these conditions can increase enzymatic activity and microbial metabolism of fecal material. Subsequent heat-catalyzed chemical reactions alter glucocorticoid metabolites, and lead to a greater affinity for the antibody used in the enzyme-linked immunoassay. For this reason, samples exposed to freezing and thawing are less likely to produce accurate measurements (Pappano et al., 2010).

By assessing sample viability in a temperate climate with significant snow cover for much of winter, we sought to further knowledge regarding the influence of environmental conditions on the duration of fecal sample viability for assaying glucocorticoid metabolites. Our 90-day study extended from midwinter to spring thaw, and suggests that midwinter fecal glucocorticoid metabolite concentrations could remain stable beyond 10 days if temperatures remain below freezing. Our ability to infer how long samples may actually remain viable, unfortunately, was compromised by an unseasonable warm up to 9.3°C on Day 16 (16 February 2017) of the exposure period. Consequently, to clarify the role of ambient winter conditions on fecal sample viability, we recommend that future field exposure studies be coupled with companion studies under controlled environmental conditions. Such a study could be designed to mimic a range of anticipated field conditions and contrasted with *in situ* results.

Midwinter thawing and refreezing produced aberrant measurements, which stabilized once freezing conditions returned. Spring thaw resulted in elevated readings likely in response to microbial activity. Consequently, four main conclusions arise from our study. First, fecal samples collected from snowpack during periods of below freezing temperatures likely provide a stable integrated index of animal condition. Second, the efficacy of fecal glucocorticoid metabolite assays is strongly dependent on environmental conditions, which should be monitored to ensure sample viability (Abaigar et al., 2010). Third, given the strong response to freeze-thaw cycles, frozen samples should be processed immediately after they are defrosted. Finally, changing climate conditions may greatly influence the utility of this method in field studies at northern latitudes if they result in fewer periods of persistent below freezing temperatures (Easterling, 2000; Walther et al, 2002; Kreyling, 2010).

Field studies involving non-invasive assessment of physiological stress in response to disturbance are becoming more prevalent, and our results underscore the importance of considering deposition environment and exposure time for measuring fecal glucocorticoid metabolites. Specifically, such measurements may be useful as indices of stress profiles of a particular population. By expanding our understanding of the limitations of this technique, field sampling protocols can be adjusted for seasonality to ensure the most accurate results, and allow for accurate monitoring of free-ranging wildlife in a changing environment.

## Appendix 1

Hormone levels can be measured from samples of bodily fluid (e.g. blood, urine or fecal extract using immunoassays). Immunoassays are designed to measure the concentration of a protein of interest, such as a hormone, and are sensitive to measure minute quantities of that hormone (Guyton and Hall, 2006).

In the early 1960s, a breakthrough technique known as radioimmunoassay (RIA) was invented to measure hormone concentrations in a fluid sample. RIA involves combining a fluid sample with a hormone-specific antibody. An appropriate amount of purified standard hormone is then added, tagged with a radioactive isotope. In order for a RIA to work, there cannot be enough antibody to bind both the tagged hormone and sample hormone (Guyton and Hall, 2006).

The hormone in the sample competes for antibody-binding sites with the tagged hormone, thus the proportion of binding by each hormone is proportional to concentration in the assay. Once binding reaches equilibrium, the antibody-hormone complex is then isolated and radioactive hormone levels are measured using a radioactive counter. High quantities of radioactive hormone bound to the antibody indicates a low concentration of the hormone in a sample, while low quantities of radioactive hormone indicate higher concentration of the hormone in a sample.

By the 1970s, enzyme-linked immunosorbent assay (ELISA) were introduced as an alternative technique for measuring hormone levels. ELISA relies on enzyme specificity coupled with sensitive enzyme assays. ELISA is not only more sensitive, but also bypasses the safety concerns associated with radioactive isotopes, as well as the need for costly measuring instruments (Beuvery et al., 1984).

ELISA involves placement of samples on a microplate coated with a hormone-specific antibody. A second antibody is introduced, binding to different sites on the hormone molecule. A third antibody, combined with an enzyme is then introduced to produce a substrate and catalyze a reaction that produces a product that can be measured with a spectrophotometer (Guyton and Hall, 2006).

Both types of immunoassay require use of standards, samples of purified hormone in different concentration, used to calculate a standard curve. This curve is then used to compute hormone concentration in a sample.

## References

[coy071C1] Abaigar T, Domené MA, Palomares F (2010) Effects of fecal age and seasonality on steroid hormone concentration as a reproductive parameter in field studies. Eur J Wildl Res 56: 781–787.

[coy071C2] Armstrong RL, Brodzik MJ (2001) Recent northern hemisphere snow extent: a comparison of data derived from visible and microwave satellite sensors. Geophys Res Lett 28: 3673–3676.

[coy071C3] Barelli C, Rovero F, Hodges K, Araldi A, Heisterman M (2015) Physiological stress levels in the endemic and endangered Udzungwa Red Colobus vary with elevation. Afr Biol 50: 23–30.

[coy071C4] Barton K (2018) MuMIn: Multi-Model Inference. R package version 1.40.4. https://CRAN.R-project.org/package=MuMIn

[coy071C5] Beehner JC, Whitten PL (2004) Modifications of a field method for fecal steroid analysis in baboons. Physiol Behav 82: 269–277. 15276788 10.1016/j.physbeh.2004.03.012

[coy071C6] Beuvery EC, Kayhty MH, Leussink AB, Kanhai V (1984) Comparison of radioimmunoassay and enzyme-linked immunosorbent assay in measurement of antibodies to *Neisseria meningitidis* group A capsular polysaccharide. J Clin Microbiol 20: 672–676. 6436314 10.1128/jcm.20.4.672-676.1984 PMC271408

[coy071C7] Bubenik GA, Bubenik AB, Brown GM, Trenkle A, Wilson DI (1975) Growth hormone and cortisol levels in the annual cycle of white-tailed deer (*Odocoileus virginianus*). Can J Physiol Pharmacol 53: 787–792. 1201483 10.1139/y75-108

[coy071C8] Creel S, Fox JE, Hardy A, Sands J, Garrott B, Peterson RO (2002) Snowmobile activity and glucocorticoid stress response in wolves and elk. Conserv Biol 16: 809–814.

[coy071C9] Dantzer B, Fletcher QE, Boonstra R, Sheriff MJ (2014) Measures of physiological stress: a transparent or opaque window into the status, management, and conservation of species? Conserv Physiol 2: cou023 doi: 10.1093/conphys/cou023. 27293644 10.1093/conphys/cou023 PMC4732472

[coy071C10] Doepker RV, Beyer DE, Donovan ML (1994) Deer population trends in Michigan’s Upper Peninsula. Michigan Department of Natural Resources, Wildlife Division, Lansing.

[coy071C11] Easterling DR (2000) Climate extremes: observations, modeling, and impacts. Science 289: 2068–2075. 11000103 10.1126/science.289.5487.2068

[coy071C13] Evans N, Narayan EJ, Hero JM (2013) Effects of natural weathering conditions on faecal cortisol metabolic measurements in the greater bilby (*Macrotis lagotis*). Austral. J Zool 61: 351–356.

[coy071C14] Guyton AC, Hall JE (2006) Textbook of medical physiology. Elsevier Saunders, Philadelphia, pp 915–916.

[coy071C15] Keppel G (1991) Design and analysis: a researcher’s handbook, 3rd ed Prentice-Hall, Inc, Englewood Cliffs, NJ, US.

[coy071C16] Koren L, Whiteside D, Fahlman A, Ruckstuhl K, Kutz S, Checkley S, Dumond M, Wynne-Edwards K (2012) Cortisol and corticosterone independence in cortisol-dominant wildlife. Gen Comp Endocrinol 177: 113–119. 22449618 10.1016/j.ygcen.2012.02.020

[coy071C17] Kreyling J (2010) Winter climate change: a critical factor for temperate vegetation performance. Ecology 91: 1939–1948. 20715613 10.1890/09-1160.1

[coy071C18] Mesa-Cruz JB, Brown JL, Kelly M (2014) Effects of natural environmental conditions on fecal glucocorticoid metabolite concentrations in jaguars (*Panthera onca*) in Belize. Conserv Physiol 2: cou39 10.1093/con-phys/cou39. PMC4732494 27293660

[coy071C19] Millspaugh JJ, Washburn BE (2003) Within sample variation of fecal glucocorticoid metabolites. Gen Comp Endocrinol 132: 21–26. 12765640 10.1016/s0016-6480(03)00061-3

[coy071C20] Millspaugh JJ, Washburn BE (2004) Use of fecal glucocorticoid metabolite measures in conservation biology research: considerations for application and interpretation. Gen Comp Endocrinol 138: 189–199. 15364201 10.1016/j.ygcen.2004.07.002

[coy071C21] Mladenoff DJ, Stearns F (1993) Eastern hemlock regeneration and deer browsing in the Northern Great Lakes region: a re-examination and model simulation. Conserv Biol 7: 889–900.

[coy071C22] Morrison SF, Forbes GJ, Young SJ, Lusk S (2003) Browse occurrence, biomass and use by white-tailed deer in a northern New Brunswick deer yard. Can J For Res 32: 1518–1524.

[coy071C23] Murray BD, Webster CR, Bump JK (2014) A migratory ungulate facilitates cross-boundary nitrogen transport in forested landscapes. Ecosystems 17: 1002–1013.

[coy071C24] Möstl E, Messmann S, Bagu E, Robia C, Palme R (1999) Measurement of glucocorticoid metabolite concentrations in faeces of domestic livestock. Zentralbl Veterinarmed A 46: 621–631. 10638300 10.1046/j.1439-0442.1999.00256.x

[coy071C25] Möstl E, Rettenbacher S, Palme R (2005) Measurement of corticosterone metabolites in birds’ droppings: an analytical approach. Ann N Y Acad Sci 1046: 17–34. 16055841 10.1196/annals.1343.004

[coy071C26] National Data Buoy Center (2017) Historical Data and Climatic Summaries, Feb 1-May 2 in 2017. http://www.ndbc.noaa.gov/station_history.php?station=pclm4. (last accessed 7 Feb 2018).

[coy071C27] National Operational Hydrologic Remote Sensing Center (2004) Snow Data Assimilation System (SNODAS) Data Products at NSIDC, Feb 1-May 2 in 2017. Boulder, Colorado USA: National Snow and Ice Data Center. Digital media. [Date accessed 6 Feb 2018].

[coy071C28] National Weather Service (2018) National Weather Service, Marquette, Michigan. http://www.crh.noaa.gov/mqt/index.php?page=climate/normals/houghton (last accessed 22 March 2018).

[coy071C29] Nelson ME, Mech LD (1981) Deer social organization and wolf predation in northeastern Minnesota. Wildl Monogr 77: 3–53.

[coy071C30] Palme R, Touma C, Arias N, Dominchin MF, Lepschy M (2013) Steroid extraction: get the best out of faecal samples. Wien Tierärztl Monatsschr 100: 238–246.

[coy071C31] Pappano DJ, Roberts EK, Beehner JC (2010) Testing extraction and storage parameters for a fecal hormone method. Am J Primatol 72: 934–941. 20623500 10.1002/ajp.20859

[coy071C45] Parnell T, Narayan EJ, Nicolson V, Martin-Vegue P, Mucci A, Hero J (2015) Maximizing the reliability of non-invasive endocrine sampling in the tiger (Panthera tigris): environmental decay and intraspecific variation in fecal glucocorticoid methabolites. Cons Phys 3: 1–7. 10.1093/conphys/cov053 PMC4778480 27293737

[coy071C32] Pinheiro J, Bates D, DebRoy S, Sarkar D, R Core Team (2017) *nlme: Linear and Nonlinear Mixed Effects Models* R package version 3.1–131, https://CRAN.R-project.org/package=nlme.

[coy071C33] R Core Team (2017) R: A language and environment for statistical computing. R Foundation for Statistical Computing, Vienna, Austria. URL https://www.R-project.org/.

[coy071C34] Sheriff MJ, Dantzer B, Delehanty B, Palme R, Boonstra R (2011) Measuring stress in wildlife: techniques for quantifying GCs. Oecologia 166: 869–887. 21344254 10.1007/s00442-011-1943-y

[coy071C35] Verme LJ (1973) Movements of white-tailed deer in Upper Michigan. J Wildl Manag 37: 545–552.

[coy071C36] Walther G, Post E, Convey P, Menzel A, Parmesan C, Beebee T, Fromentin J, Hoegh-Guldberg O, Bairlein F (2002) Ecological responses to recent climate change. Nature 416: 389–395. 11919621 10.1038/416389a

[coy071C37] Washburn BE, Millspaugh JJ (2002) Effects of simulated environmental conditions on glucocorticoid metabolite measurements in white-tailed deer feces. Gen Comp Endocrinol 127: 217–222. 12225762 10.1016/s0016-6480(02)00056-4

[coy071C38] Wikelski M, Cooke SJ (2006) Conservation physiology. Trends Ecol Evol 21: 1–9. 16701468 10.1016/j.tree.2005.10.018

[coy071C39] Wilkening JL, Ray C, Varner J (2016) When can we measure stress noninvasively? Postdeposition effects on fecal stress metric confound a multiregional assessment. Ecol Evol 6: 502–513. 26843934 10.1002/ece3.1857 PMC4729247

